# Prevalence and risk factors for depression in factitious disorder: a systematic review

**DOI:** 10.3389/fpsyt.2024.1355243

**Published:** 2024-04-26

**Authors:** Carla Comacchio, Delia Manuela Misca, Riccardo Bortoletto, Alvisa Palese, Matteo Balestrieri, Marco Colizzi

**Affiliations:** ^1^ Unit of Psychiatry, Department of Medicine (DMED), University of Udine, Udine, Italy; ^2^ School of Nursing, Department of Medicine (DMED), University of Udine, Udine, Italy; ^3^ Department of Psychosis Studies, Institute of Psychiatry, Psychology and Neuroscience, King’s College London, London, United Kingdom

**Keywords:** factitious disorder, depression, prevalence, risk factors, comorbidity

## Abstract

**Objective:**

Factitious disorder is characterized by a pattern of abnormal behavior in which patients deliberately produce, falsify, or exaggerate physical and/or psychological symptoms that have no, or little, organic basis, to assume the sick role. In the context of a factitious disorder, depression can be both a feigned disease and an associated comorbidity. We performed a systematic review to provide an overview of the relationship between factitious disorder and depression, describe the prevalence of depression in factitious disorder, and identify factors that can contribute to the development of depression in patients suffering from factitious disorder.

**Methods:**

A literature search was performed using the electronic databases PubMed, EMBASE and Cochrane Library following the Preferred Reporting Items for Systematic Reviews and Meta-Analyses (PRISMA) guidelines. Studies were eligible for inclusion in this review if they investigated factitious disorder or Munchausen Syndrome with comorbid depression.

**Results:**

Depression was found to be highly prevalent in factitious disorder, affecting around 30% of the samples. Risk factors for depression in factitious disorder included having suffered from childhood and adulthood traumatic experiences and having a history of psychosocial problems.

**Conclusion:**

The treatment of factitious disorder is challenging and requires a multidisciplinary team approach. Given the high levels of depression in patients with factitious disorder, we recommend to always screen for depression once a factitious disorder is diagnosed.

## Introduction

Factitious disorder is characterized by a pattern of abnormal behavior in which patients deliberately produce, falsify, or exaggerate physical and/or psychological symptoms that have no, or little, organic basis, to assume the sick role (1). Factitious disorder can be misdiagnosed as conversion disorder, but in conversion disorder the production of physical and/or psychological symptoms is unconscious, whereas in factitious disorder this production is conscious. Factitious disorder can also be imposed on other people, when the perpetrator actively harms his victims in order to make them ill. In such a case, the disorder is also called Munchausen Syndrome by proxy. Factitious disorder imposed on another can involve a dependent adult, an elderly person, or a child as a victim and, in the latter case, it is a form of childhood abuse (2, 3). Factitious disorder was first described by the British psychiatrist Asher in 1951, and named after Baron Hieronymous Karl Friedrich von Münchausen (1720–1791), a German officer who was known for telling invented and unbelievable stories about himself and his life (4) The American Psychiatric Association first included factitious disorder in Diagnostic and Statistical Manual of Mental Disorders (DSM) III: Diagnostic and Statistical Manual of Mental Disorders in 1980 (5). However, despite decades have passed since the inclusion of factitious disorder in the DSM manual, its incidence remains controversial (6, 7). According to DSM-5, factitious disorder in hospital settings is estimated to be present in 1% of individuals (1). Skin alteration (i.e., ulcers, dermatitis artefacta, hyperkeratosis) is the most common presentation of factitious disorder (8, 9), but factitious disorder appears to be common also in neurological settings, where it represents up to 30% of neurologist consultations (10).

In the context of factitious disorder, depression can be both a feigned disease and an associated comorbidity. However, since most patients with factitious disorder reject psychiatric consultation, its real prevalence is likely underestimated (6, 11). As a consequence, only a few patients with factitious disorder that also present with depression receive an adequate psychiatric diagnosis (12). Moreover, it is important to note that not all patients with factitious disorder suffer from depression, and literature on risk factors for depression development in factitious disorder patients is scarce and has never been put into a congruent frame. Based on these premises, the present study aimed to: 1. Provide an overview of the relationship between factitious disorder and depression; 2. Identify the prevalence of depression in factitious disorder; 3. Identify factors that can contribute to the development of depression in patients suffering from factitious disorder.

## Methods

**Figure 1:**
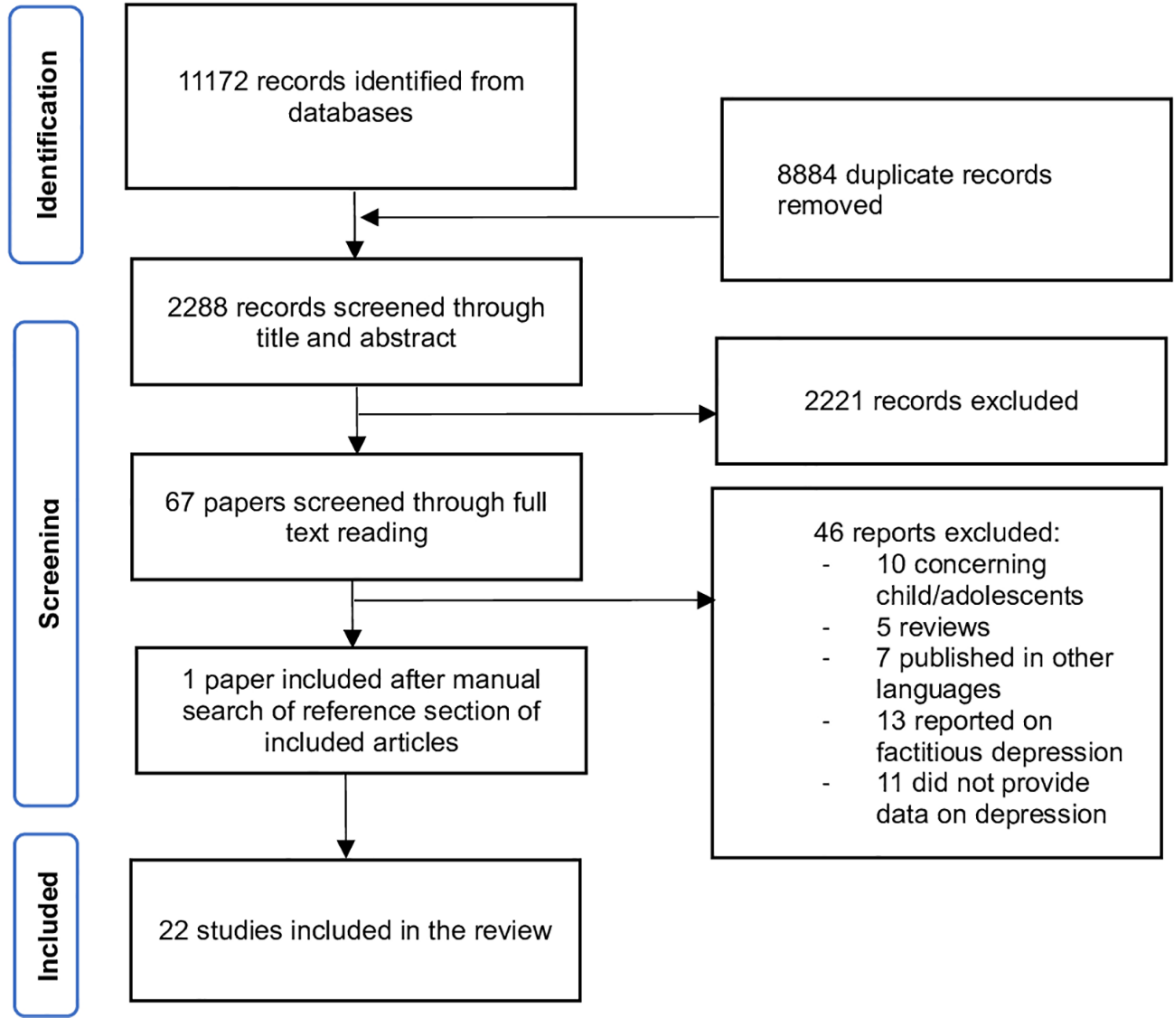
Flow diagram of the screening process according to PRISMA (13).

The review followed the Preferred Reporting Items for Systematic Reviews and Meta-Analyses (PRISMA) guidelines (13). A literature search was performed using the electronic databases PubMed, EMBASE and Cochrane Library, using a combination of the following MESH terms: “factitious disorder”, “Munchausen Syndrome”, “depression”, “depressive disorder” and “depressive episode. The search was conducted on December 9^th^, 2022. Studies were eligible for inclusion in this review if they investigated factitious disorder imposed on self or imposed on another with comorbid depression. Only original papers published in English, French or Italian in peer-reviewed journals were accepted for inclusion. No predefined time window for the study search was adopted, to be the most inclusive as possible. By using a three-step screening approach, articles were screened through title, abstract, and full-text reading, if needed. Studies were excluded if they (i) reported on children and adolescents; (ii) provided mainly commentary or proposed guidelines; (iii) did not assess depression in factitious disorder; or (iv) reported on factitious depression. The screening and data extraction was done manually. Publication data screening and extraction were performed following a 2-step selection process (conventional double-screening) conducted by two reviewers independently of each other (CC and DMM). In the rare instances of discrepant screening, a consensus was reached through discussion with a third senior clinical researcher (MC). Further research evidence, gathered outside of the search or identified through manual search of the reference section of the included articles, was reported if considered appropriate by researchers. By applying a flexible approach, other articles that were deemed to cover prominent related topics were also searched by accessing grey literature and/or screening the reference lists of the eligible studies, to provide a more comprehensive overview (See **Figure 1** – flow chart). The following information was extracted from the included studies: Study ID (including authors, year of publication and country in which the study was conducted), study design characteristics (including study type, number of patients and patients’ sex and age), brief description of symptoms presentation, diagnostic tools used and risk factors for depression.

## Results

A total of 2288 articles were identified and cross-checked by two researchers. By using a three-step screening approach, titles, abstracts, or full texts of all records were screened against the inclusion and exclusion criteria. A total of 22 articles were included, consisting of *n* = 18 case reports (2, 6, 12, 14–28) and *n =* 4 cohort studies (9, 11, 29, 30).

The studies were conducted in 13 countries, with 33% of them being performed in the United States (US), 33% in the European Union (EU), and 33% in Turkey, Morocco, Canada, and India, by involving from 1 to 60 patients, mainly female. The most common presentation of factitious disorder was skin lesions (*n* = 11) and hypoglycemia (*n* = 2). Beyond this, there was a wide range of presentations, such as factitious mourning, Acquired immune deficiency syndrome (AIDS), cancer, Cushing syndrome, vomiting, and anaphylaxis.

### Diagnostic procedures

In all studies included the diagnosis of factitious disorder was made after exclusion of any medical condition, prolonged clinical examination, and detailed history collection. To define the presence of depression, we adhered to the criteria adopted in the individual studies. Depression was diagnosed after clinical examination (*n* = 13) or instrumental assessment (*n* = 6). Two studies did not specify how depression was diagnosed. Among diagnostic tools, Minnesota Multiphasic Personality Inventory (MMPI) was used in *n* = 2 studies; Beck Depression Inventory (BDI) and Hospital and Anxiety Depression Scale (HADS) were used in *n* = 1 study; projective tests such as the Rorschach test (31), the Rosenzweig Picture-Frustration Test (32) and the Rotter Sentence Completion Test (33) were used in *n* = 2 studies. One study reported generic “psychological test” without any further explanation. The Rosenzweig Picture-Frustration Test is a projective technique for the assessment of frustration tolerance and of how a person reacts to conflict situations; The Rotter Sentence Completion Test is a sentence completion test intended to detect psychological maladjustment. Intelligence Quotient (IQ) was investigated in *n* = 2 studies (see **Table 1**). Factitious disorder imposed on another was diagnosed in *n* = 2 studies and involved mothers in their post-partum (2, 22).

**Table 1:** Characteristics of the included studies.

Author, year	Country	Nr of patients Nr of depressed	Sex, age	Diagnostic tools	Type of factitious disorder	Risk factors	Psychiatric treatment
Cohort studies							
Phillips, 1983 (30)	USA	20 – 7 depressed	14 M 7 F Mean age: 32	IQ test MMPI	Mourning	History of psychiatric disorder Drug or alcohol abuse Intellectual disability Personality disorder Being single or divorced	Antidepressant
Haenel, 1984 (29)	CH	60 – 23 depressed	5 M; 55 F Mean age: 37	CPT RPFT	Skin lesions	History of psychiatric disorder Personality disorder Childhood trauma Adulthood trauma	–
Fliege, 2009 (11)	D	19 - 4 depressed	45 M 149 F Mean age: 37	BDI HADS PSQ LOT	Various types of self-harm		–
Mohandas, 2013 (9)	UK	28 – 11 depressed	4 M 24 F Mean age: 37	Clinical examination	Skin lesions	History of psychiatric disorder Life stressors Drug or alcohol abuse Childhood trauma Being single or divorced	Antidepressant, psychotherapy
Case reports							
Earle, 1986 (12)	USA	1	F, 27	Clinical examination	Skin lesions	History of psychiatric disorder Childhood trauma Adulthood trauma Being divorced Mourning	Antidepressant
Silva, 1989 (25)	USA	1	F, 28	MMPI	AIDS	Personality disorder	Antidepressant
Feldman, 1991 (18)	USA	1	F, 35	Psychological test	Cancer	Relationship problems	Antidepressant
Cizza, 1996 (16)	USA	2	F, 44 F, 32	Clinical examination	Cushing syndrome	History of psychiatric disorder Drug or alcohol abuse Childhood trauma	–
Moszkowicz, 1998 (22)	DK	1	F, -	WAIS Rorschach RSCT	FDIOA	History of psychiatric disorder Personality disorder	–
Waickus, 1998 (27)	USA	1	F, 39	Clinical examination	Hypoglicemia	–	–
Gojer, 2000 (19)	CAN	1	F, 28	Clinical examination	FDIOA	History of psychiatric disorder	Psychopharmacotherapy, psychotherapy
Tosun, 2005 (26)	TR	1	M, 21	Clinical examination	Subcutaneous emphysema	Drug or alcohol abuse	Psychopharmacotherapy
Oh, 2005 (24)	UK	1	F, 35	Clinical examination	Skin lesions	History of psychiatric disorder	Antidepressant
Lee, 2010 (21)	TW	1	F, 29	Clinical examination	Subcutaneous emphysema	History of psychiatric disorder Family problems Drug or alcohol abuse Adulthood trauma	Psychopharmacotherapy
Kucuker, 2010 (2)	TR	1	F,32	Clinical examination	FDIOA (hypoglicemia)	Family problems	Antidepressant
Borojeni, 2011 (20)	IR	1	F, 34	Clinical examination	Vomiting and abdominal pain	Childhood abuse Family problems Mourning	Antidepressant
Chiriac, 2014 (15)	RO	2	F, 77 F, 61	Clinical examination	Skin lesions	–	Antidepressant
Giuliodori, 2014 (6)	I	2	M, 76 F, 40	Clinical examination	Skin lesions	History of psychiatric disorder Personality disorder Adulthood trauma Mourning	Antidepressant
Zinoun, 2015 (28)	MA	1	F, 27	–	Skin lesions	History of psychiatric disorder Family problems Mourning	Antidepressant, psychotherapy
Nolkha, 2017 (23)	IND	1	F, 22	Clinical examination	Skin lesions	Life stressors Childhood trauma	Antidepressant
El Amraoui, 2018 (17)	MA	1	M, 22	Clinical examination	Skin lesions	Family problems	–
Khanal, 2021 (14)	USA	1	F, 23	–	Anaphilaxis	History of psychiatric disorder Family problems	Antidepressant, ECT

AIDS, Acquired Immune Deficiency Syndrome; BDI, Beck’s Depression Inventory; CAN, Canada; CH, Swiss; CPT, Colour Pyramid Test; D, Germany; DK, Denmark; ECT, Electro-Convulsive Therapy; F, Female; FDIOA, Factitious Disorder Imposed on Another; HADS, Hospital Anxiety and Depression Scale; I, Italy; IND, India; IQ, Intelligence Quotient; IR, Iran; LOT, Life Orientation Test; M, Male; MA, Morocco; MMPI, Minnesota Multiphasic Personality Inventory; PSQ, Perceived Stress Questionnaire; RO, Romania; RPFT, Rosenzweig Picture-Frustration Test; RSCT, Rotter’ Sentence Completion Test; TR, Turkey; TW, Taiwan; UK, United Kingdom; USA, United States of America; WAIS, Wechsler Adult Intelligence Scale.

### Prevalence

Prevalence of depression in factitious disorder was reported for all the four cohort studies. Phillips (30) reported on 20 patients with factitious mourning (defined as the falsely reported death of loved ones), 7 of which (35%) were later diagnosed as having depression. Haenel (29) analyzed 60 cases of factitious dermatitis, 23 of which (38%) showed symptoms of depression. Fliege (11) analyzed 19 cases of factitious disorder referring to a department of psychosomatic medicine and found a 15.8% prevalence of depression and a 26.3% prevalence of anxiety. Finally, Mohandas (9) found that 10 patients out of a cohort of 28 patients with factitious dermatitis were suffering from depression (36%).

### Factitious disorder and depression

Patients with factitious disorder and depression displayed the classic features of depression: tendency to weep, feelings of guilt, loss of interest in daily activities, and loss of concentration (25, 28). Also, they reported insomnia, suicidal thoughts, loss of appetite, and fatigue (15, 17, 19, 25, 28).

Depression was treated with antidepressant in *n* = 16 studies. Add-on treatment included psychotherapy (*n* = 2) and electroconvulsive therapy (*n* = 1). In all cases, antidepressant treatment led to an improvement of both factitious and depressive symptoms (See **Table 1**).

### Risk factors

Risk factors for factitious disorder comorbid with depression were reported in *n* = 20 studies. They included: history of childhood trauma, mourning, recent divorce or severe family problems, specific psychological traits such as high levels of psychological tension and scarce tolerance to frustration, history of psychiatric disorders, drug abuse and intellectual disability (see **Table 1**).

## Discussion

This review found depression to be highly prevalent in factitious disorder, affecting around 30% of the samples. This result provides support for an association between factitious disorder and mood disturbance (34). According to evidence gathered in this review, signs and symptoms of depression in factitious disorder are identical to those expressed by patients with depression who do not have a factitious disorder. For this reason, depression among factitious disorder patients is expected to be easily identified by an expert psychiatrist. Diagnostic tools for depression can be used, as well as projective tests. Projective tests can be especially helpful when factitious depression is suspected. The theoretical basis for use of projective measures is that in the absence of specific instruction or highly directive stimuli, people will have only their own internal resources available for managing the demands of the test. Thus, they will project their own internal psychological functioning onto the test stimuli (31).

Results of our review suggest that patients with factitious disorder and comorbid depression who receive antidepressant treatment improve both in factitious and depressive symptoms. Therefore, early detection and treatment of depressive symptoms in this population appears to be crucial also for the management of the factitious disorder. Factitious disorder diagnosis is often problematic due to the nature of the disorder that leads clinicians to focus more on somatic symptoms rather than on psychological problems, at least at first. The exaggerated, atypical, and contradictory presentation of factitious disorder symptoms is likely to lead clinicians to perform unnecessary diagnostic tests and invasive diagnostic procedures and to prescribe unnecessary treatments and needless hospitalization, also to avoid exposure to malpractice litigation (35, 36). Patients’ seeking attitude towards procedures and treatment goes along with clinicians’ fears of litigation, which can result in important delays in factitious disorder diagnosis. Moreover, the focus on somatic complains and the waiting for procedure results may lead clinicians to underestimate levels of emotional distress in patients with factitious disorder (6). Indeed, it is likely that there is a significant window of time in which depression in factitious disorder goes undetected. Importantly, delays in depression treatment in patients with factitious disorder have been related to poor prognosis and high risk of chronicity of both depressive and factious symptoms (37).

Reported risk factors for depression in factitious disorder overlap with risk factors for factitious disorder with regard to childhood trauma and history of psychiatric disorder. The association between childhood adversities and the subsequent development of depression in adult life has been extensively studied (38). Childhood abuse involves experiences of being rejected, degraded, terrorized, isolated or teased. When childhood abuse is perpetrated by caregivers, it affects secure attachment, which can lead to the development of distorted and negative internal working models of the self and the others (39). Childhood abuse and insecure attachment are also linked to alexithymia, which is the inability to express and regulate emotions. Alexithymia is often found across several mental disorders, including depression. Further, such inability to recognize others’ emotions and to properly express one’s own emotions can lead to the development of somatic symptoms. The production of somatic symptoms, either conscious or unconscious, may thus be functional to avoid trauma-related symptoms. People exposed to childhood traumatic experiences are more likely to develop emotional distress compared to non-exposed. Emotional distress may lead to emotional fragility, feelings of insecurity, social isolation, low self-esteem, and loneliness intolerance, that also continue into adult life, leading to the development of a factitious disorder with comorbid depression (6, 28). With regard to having a history of psychiatric disorder, it is known that some psychiatric disorders, such as bipolar and personality disorders, are at high risk to present with depressive episodes. For this reason, it is likely that having a factitious disorder and a history of psychiatric disorder increases the odds of developing depression. Instead, specific risk factors for depression in factitious disorder seem to include having suffered from adulthood traumatic experiences, especially mourning. Under this perspective, factitious disorder symptoms may act as a try to avoid the foster of complex situations related to traumatic experiences underlying depression.

### Strengths and limitations

To our knowledge, this is the first review exploring the interplay between depression and factitious disorder. Following PRISMA guidelines, our systematic review described characteristics of depression in factitious disorder. We found that depression is highly prevalent in factitious disorder, affecting 1:3 patients and we identified and discussed specific risk factors for depression and factitious disorder. However, the main limitation of the study is that most evidence came from case reports, whose methodology is not always robust. Although in the hierarchy of evidence-based medicine, case reports do not top the list, the hypothesis generated from them may be appealing, leading to physiological studies and clinical trials (40). In addition, there was high heterogeneity in terms of screening tools for depression and assessment procedures. Also, even though the systematic review followed the PRISMA statement, no review protocol was registered. Lastly, a significant proportion of articles included in the present systematic review are older than 5 years. This could reflect a progressive reduction of interest in the topic, possibly accelerated by the switch towards psychological themes related to the Coronavirus Disease 2019 (COVID-19) pandemic in recent years.

## Conclusions

Given the high prevalence of depression in factitious disorder, a multidisciplinary team approach in cooperation with mental health professionals appears to be essential for the management of these patients. However, this field of research is still sparse and mainly based upon case-report studies, suggesting the need to increase research in this area with more robust investigations.

## Data availability statement

The original contributions presented in the study are included in the article. Further inquiries can be directed to the corresponding author.

## Author contributions

CC: Conceptualization, Data curation, Investigation, Methodology, Resources, Validation, Visualization, Writing – original draft, Writing – review & editing. DMM: Conceptualization, Data curation, Investigation, Resources, Validation, Visualization, Writing – original draft, Writing – review & editing. RB: Conceptualization, Data curation, Investigation, Resources, Validation, Visualization, Writing – review & editing. AP: Conceptualization, Methodology, Resources, Supervision, Validation, Visualization, Writing – review & editing. MB: Conceptualization, Methodology, Project administration, Resources, Supervision, Validation, Visualization, Writing – review & editing. MC: Conceptualization, Methodology, Project administration, Resources, Supervision, Validation, Visualization, Writing – review & editing.
